# Assessing the effect of varying sequence length on DNA barcoding of fungi

**DOI:** 10.1111/j.1471-8286.2007.01698.x

**Published:** 2007-05-01

**Authors:** XIANG JIA MIN, DONAL A HICKEY

**Affiliations:** Department of Biology, Concordia University 7141 Sherbrooke West, Montreal, Quebec, Canada H4B 1R6

**Keywords:** biodiversity, COI, cytochrome oxidase, fungi, taxonomy

## Abstract

DNA barcoding shows enormous promise for the rapid identification of organisms at the species level. There has been much recent debate, however, about the need for longer barcode sequences, especially when these sequences are used to construct molecular phylogenies. Here, we have analysed a set of fungal mitochondrial sequences — of various lengths — and we have monitored the effect of reducing sequence length on the utility of the data for both species identification and phylogenetic reconstruction. Our results demonstrate that reducing sequence length has a profound effect on the accuracy of resulting phylogenetic trees, but surprisingly short sequences still yield accurate species identifications. We conclude that the standard short barcode sequences (∼600 bp) are not suitable for inferring accurate phylogenetic relationships, but they are sufficient for species identification among the fungi.

## Introduction

DNA barcoding has been promoted as a potentially powerful method for the efficient, accurate and high throughput assignment of unknown specimens to known species. It has been tested in a number of animal taxa, including birds (Hebert *et al*. 2004b), fishes (Ward *et al*. 2005), and Lepidopteran insects (Hebert *et al*. 2004a; Hajibabaei *et al*. 2006a). In all of these studies, a 648-bp region of the mitochondrial cytochrome *c* oxidase I (COI or cox1) has been evaluated and then used as a ‘biological barcode’ for species identification. In flowering plants, the nuclear internal transcribed spacer region (ITS) and the plastid trnH-psbA intergenic spacer have been proposed as potential DNA barcodes (Kress *et al*. 2005). Reliable DNA barcodes would provide a practical, standardized, species-level identification tool for biodiversity assessment and ecological studies. An international consortium of major natural history museums, herbaria and other organizations has launched an ambitious project, the ‘Barcode of Life Initiative’, to promote using barcodes for identifying the estimated 10 million species on earth (http://barcoding.si.edu/index.htm) (Savolainen *et al*. 2005).

Although DNA barcodes show great promise for species identification, their use in molecular phylogenetics is more problematic. It has recently been shown (Hajibabaei *et al*. 2006b) that phylogenetic trees constructed from short barcode sequences, although approximately reflective of accepted phylogenetic relationships, have low statistical support at many of the internal nodes and can seriously misrepresent some of the branching pattern.

The goal of this study was to assess the efficiency of DNA barcoding for both the classification of unknown specimens at the species level and for phylogenetic reconstruction. Since we were particularly interested in the effect of varying barcode sequence length, we began our study with sequences that were much longer than conventional DNA barcodes and then systematically reduced this length.

We chose fungal mitochondrial sequences as our test data set. This enabled us not only to monitor the effect of decreasing sequence length on barcoding efficiency, but it also allowed us to compare the sequence diversity of animal and fungal barcode regions. Fungi are an important group of organisms, not only because of their roles in ecosystem function but also because of their influence on human health and industrial processing. The kingdom Fungi encompasses a broad range of taxa, morphologies, ecologies and life-history strategies (Mueller *et al*. 2004). Among the 1.5 million species that was hypothesized by Hawksworth (1991), less than 5% have been described. Thus, there is an urgent need for efficient methods of fungal species identification.

Our strategy was to begin our analysis with the concatenated sequences of five mitochondrial genes that are common to all fungi. This provided us with more than 5 kb of sequence for each species. We then proceeded to look at the results for individual genes and, eventually, for gene fragments of decreasing length.

## Materials and methods

### DNA sequence retrieval

We examined all the protein-coding genes of completely sequenced mitochondrial genomes in fungi (http://www.ncbi.nlm.nih.gov/genomes/ORGANELLES/fu.html) and found there are only five protein-coding genes common in all fungal mitochondrial genomes. These five genes encode cytochrome *c* oxidase subunit 1 (cox1), cytochrome *c* oxidase subunit 2 (cox2), cytochrome *c* oxidase subunit 3 (cox3), cytochrome *b* (cob), and ATP synthase subunit 6 (ATP6).

Both the protein-coding DNA (cDNA) and the predicted amino acid sequences from these five genes were retrieved from GenBank, comprising 31 fungal species including 27 Ascomycota, 3 Basidiomycota and 1 Chytridiomycota, which was used as an outgroup. Among these 31 species, 29 have a complete mitochondrial genome sequence available and the other two species do not have a complete mitochondrial genome sequence but have partial genomic sequences encoding the five genes of interest. The species names, their GenBank Accession nos and the phyla were listed in Table 1.

**Table 1:** Fungal species and their genome or gene sequence accession nos from which the protein sequences and protein-coding DNA sequences of cox1, cox2, cox3, cob and ATP6 were retrieved

Species	Abbreviation	GenBank Accession nos
Ascomycota		
*Ashbya gossypii*	*As. goss.*	NC_005789
*Aspergillus niger*	*As. nige.*	NC_007445
*Aspergillus tubingensis*	*As. tubi.*	NC_007597
*Candida albicans*	*Ca. albi.*	NC_002653
*Candida glabrata*	*Ca. glab.*	NC_004691
*Candida metapsilosis*	*Ca. meta.*	NC_006971
*Candida orthopsilosis*	*Ca. orth.*	NC_006972
*Candida parapsilosis*	*Ca. para.*	NC_005253
*Candida stellata*	*Ca. stel.*	NC_005972
*Epidermophyton floccosum*	*Ep. floc.*	NC_007394
*Fusarium oxysporum*	*Fu. oxyp.*	AY945289
*Hanseniaspora uvarum*	*Ha. uvar.*	NC_007780
*Kluyveromyces lactis*	*Kl. lact.*	NC_006077
*Kluyveromyces thermotoloerans*	*Kl. ther.*	NC_006626
*Lecanicillium muscarium*	*Le. musc.*	NC_004514
*Penicillium marneffei*	*Pe. marn.*	NC_005256
*Pichia canadensis*	*Pi. cana.*	NC_001762
*Podospora anserina*	*Po. anse.*	NC_001329
*Saccharomyces castellii*	*Sa. cast.*	NC_003920
*Saccharomyces cerevisiae*	*Sa. cere.*	NC_001224
*Saccharomyces servazzii*	*Sa. serv.*	NC_004918
*Schizosaccharomyces japonicus*	*Sc. japo.*	NC_004332
*Schizosaccharomyces octosporus*	*Sc. octo.*	NC_004312
*Schizosaccharomyces pombe*	*Sc. pomb.*	NC_001326
*Yarrowia lipolytica*	*Ya. lipo.*	NC_002659
*Aspergillus nidulans*	*As. nidu.*	X00790, X15411, X06960, AH001255, X01507
*Trichophyton rubrum*	*Tr. rubr.*	X65223, X88896, Y18476
Basidiomycota		
*Crinipellis perniciosa*	*Cr. pern.*	NC_005927
*Cryptococcus neoformans* var. *grubii*	*Cr. neof.*	NC_004336
*Schizophyllum commune*	*Sc. comm.*	NC_003049
Chytridiomycota		
*Allomyces macrogynus*	*Al. macr.*	NC_001715

### Sequence analysis

For each of the five genes, the predicted amino acid sequences were aligned using the clustalw algorithm (Chenna *et al*. 2003) contained within mega3 software package (Kumar *et al*. 2004). The poorly aligned terminal regions of each sequence were removed manually and the aligned portions were then concatenated. Then the concatenated sequences from the five genes were used to construct a phylogenetic tree using the neighbour-joining (NJ) method (Saitou & Nei 1987), also contained within the mega3 package. In addition to the trees based on protein sequences, we also constructed neighbour-joining trees based on the DNA sequences of the coding regions of the five genes. These DNA sequences were aligned using the protein alignment as a guide. The same methods were used to construct trees for the individual genes and for the gene fragments. For the DNA barcode regions, we chose the same segment as has been used in the recent lepidopteran barcoding study (Hajibabaei *et al*. 2006a). For the construction of the protein trees, we used the Poisson Correction model (Nei & Kumar 2000) and for the DNA-based trees, we used the Kimura 2-parameter (K2P) model (Kimura 1980). Bootstrap analyses were based on 1000 replicates.

We also compared the NJ-tree topology constructed with the concatenated coding DNA sequences of the five conserved genes that were aligned either by using clustalw directly or using protein alignment as a guide. As we found that protein-guided DNA sequence alignment was more accurate, then we used these aligned five gene-DNA sequences and DNA barcode sequences and compared the trees constructed by different methods including NJ with K2P model, Jukes-Cantor model, Tajima-Nei model and LogDet model (Kumar *et al*. 2004); maximum parsimony with close-neighbour-interchange (CNI) search method and min-mini heuristic search method (Kumar *et al*. 2004); and maximum likelihood (Guindon *et al*. 2005).

## Results

**Fig. 1:**
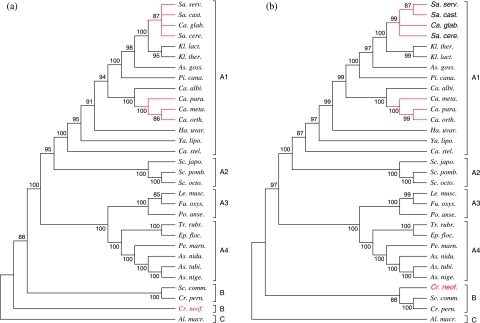
NJ trees based on relatively long mitochondrial sequences. Panel a, this tree was constructed with the concatenated cDNA sequences from five mitochondrial genes (see Methods). Panel b, this shows an NJ tree based on the predicted amino acid sequences of the same five genes. Areas where the two trees differ are highlighted in red. Clades are labelled as follows: A1, Ascomycota Saccharomyces; A2, Ascomycota Schizosaccharomyces; A3, Ascomycota Sordariomycetes; A4, Ascomycota Eurotiomycetes; B, Basidiomycota; C, Chytridiomycota.

The phylogenetic trees based on the concatenated sequences — both DNA and amino sequences — from all five genes are shown in Fig. 1. The resulting tree topologies are in good agreement with accepted fungal phylogeny (Bullerwell *et al.* 2003a, b; Kouvelis *et al*. 2004), and the majority of the internal nodes are supported by bootstrap analysis. For example, in both the DNA and protein trees (shown in Fig. 1a, b, respectively) not only were all of the species within the Ascomycota clustered together, but also the four major subdivisions within the Ascomycota phylum were resolved with high bootstrap support (bootstrap values of 87–100%). Likewise, the three species of Basidiomycetes formed a monophyletic cluster in the protein tree. In the DNA tree (Fig. 1b), however, *Cryptococcus neoformans* was not grouped with the other two Basidiomycete species. A number of other differences between the DNA-based and protein-based trees are highlighted in Fig. 1. Overall, this result shows that while long sequences can produce generally dependable phylogenies, some discrepancies remain. These discrepancies may be due to either a systematic bias in the evolution of the sequences themselves (e.g. Foster & Hickey 1999) or due to the limitations of the neighbour-joining tree-building algorithm (Saitou & Nei 1987).

**Fig. 2:**
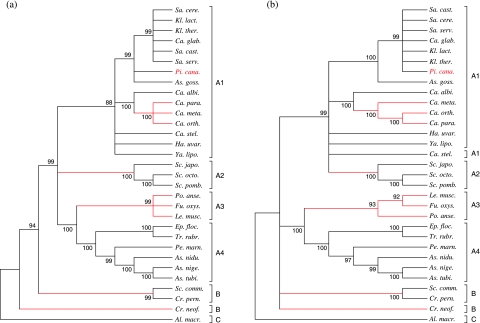
NJ trees based on the sequence of a single mitochondrial gene, cox1. Panel a shows the DNA-based tree and Panel b shows the protein tree. Differences in topology between the two trees are highlighted in red. Clades are labelled as in Fig. 1.

Having established the level of reliability of trees based on more extensive sequence data, we then proceeded to repeat the tree-building exercise using shorter and shorter sequences. First, we constructed DNA- and protein-based trees for each of the five individual genes and we compared the results to the ‘standard’ that we had established with the concatenated sequences. As expected, for all the five genes, the trees constructed with either DNA or protein sequences showed substantial decreases in the bootstrap support values, with many nodes falling below our cut-off value of 85%. We also observed numerous topological discrepancies in trees with shorter sequences in comparison with the accepted phylogenetic tree. The results for the COI gene are shown in Fig. 2 and the results for the other four genes are shown in Fig. S1–S4, Supplementary material. As can be seen in Fig. 2, the reduction in sequence length (from approximately 5 kb for the concatenated sequences to approximately 1.5 kb for the COI gene) reduces the statistical support for many of the internal nodes, although we maintain the correct clustering of the major groups, with the exception of *C. neoformans* which still branches separately from the other Basidiomycetes. A similar loss of phylogenetic signal was observed when the results were obtained for other individual genes. For example, using cox2 sequences only, the species in Schizosaccharomyces were incorrectly placed in the DNA tree (Fig. S1, Supplementary material), and when using cox3 sequences only, the phylogenetic relationships among the species in the Schizosaccharomyces were not resolved in the protein tree (Fig. S2, Supplementary material). The cob or ATP6 sequences, used alone, yielded the most poorly resolved trees. In this case, for example, the species within the Eurotiomycetes formed two clusters that were intermixed with the Sordarimycetes (Fig. S3, S4, Supplementary material). It is important to note, however, that despite the fact that the shorter, individual gene sequences cannot resolve many of the internal nodes in the tree, all of them can still resolve the terminal nodes, that is they can discriminate between individual species.

The fact that the COI gene provides the best-supported trees among the individual genes suggests that perhaps its rate of molecular evolution differs significantly from that of the other four genes. In order to investigate this possibility, we calculated the average genetic distances for each of the five genes (see Table 2). From the results, we can see that although COI shows more conservation than other genes at the protein level, the genetic distances at the DNA level are comparable to those for the cytochrome *b* and cytochrome oxidase 2 genes. The reason for the greater reliability of the COI-based trees is not due to different rates of sequence divergence but simply due to a difference in sequence length (see Table 2). Thus, this provides further confirmation that decreasing sequence length leads to decreased statistical support for the internal nodes in the tree.

**Table 2:** Overall genetic distances of cox1, cox2, cox3, cob, ATP6 and barcode DNA and protein sequences from the 31 fungal species. The DNA distance was based on Kimura 2-parameter model and the protein distance was based on Poisson Correction model. The standard errors were calculated based on 1000 bootstrap replicates

	Protein coding DNA length	DNA	Protein
cox1	1615 ± 36	0.397 ± 0.011	0.405 ± 0.018
cox2	755 ± 23	0.409 ± 0.017	0.463 ± 0.033
cox3	809 ± 9	0.495 ± 0.017	0.608 ± 0.035
cob	1161 ± 11	0.367 ± 0.017	0.429 ± 0.021
ATP6	770 ± 29	0.532 ± 0.019	0.667 ± 0.038
Barcode	599 ± 2	0.390 ± 0.020	0.403 ± 0.027

**Fig. 3:**
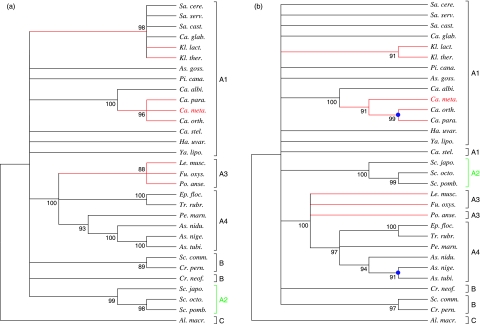
NJ trees based on the barcode region of the cox1 gene. Panel a shows the tree based on the barcode DNA sequence (∼600 bp), while Panel b shows the tree based on the predicted amino acid sequences. Differences in topology between the two trees are highlighted in red. Clades are labelled as in Fig. 1. • indicates the amino acid sequences are identical in the two species on that branch.

Our next step was to decrease the sequence length still further, to coincide with the fragment of the COI gene that has been used for DNA barcoding in animals. We then extracted a potential barcode region of 600 nt from each cox1 cDNA sequence using pre-aligned protein sequences as a guide, and we used these sequences to construct a neighbour-joining tree. The results are shown in Fig. 3. As we expected, the bootstrap support for the internal nodes in the tree is reduced further, although some phylogenetic signal remains (see Fig. 3a, b, Fig. S5, Supplementary material). The drop of the bootstrap support in the deep nodes in the tree was further confirmed with other phylogenetic tree construction methods (Fig. S6, Supplementary material). Despite this further loss of phylogenetic signal, however, the short DNA barcode sequences can still separate all of the fungal species (Fig. 3a). In contrast to this, the amino acid sequences from the barcode region are not as efficient in resolving species differences. For example, the two *Candida* species, *C. parapsilosis* and *C. orthopsilosis* were indistinguishable at amino acid level (Fig. 3b) but were distinguished at DNA level (Fig. 3a). The greater resolving power of the DNA sequences is because most of the substitutions at the species level occur at synonymous sites.

Since the short DNA sequences that correspond to the barcode region can still resolve fungal species (although they cannot resolve many of the deeper nodes in the phylogeny), we wished to explore the effect of further reducing the sequence length, even beyond the standard barcode length. The use of such very short sequences might be necessary in the case of degraded DNA template or when there are problems in polymerase chain reaction (PCR) amplification (Hajibabaei *et al*. 2006c). When we used sequences of 300 nt (corresponding to half the standard barcode length) we found a further reduction in the number of resolved internal nodes in the tree (see Fig. S7, Supplementary material). Nevertheless, nearly all of the species can still be resolved with these short, 300 nt ‘mini’ barcodes at the DNA level, consistent with the results reported by Hajibabaei *et al*. (2006c).

**Fig. 4:**
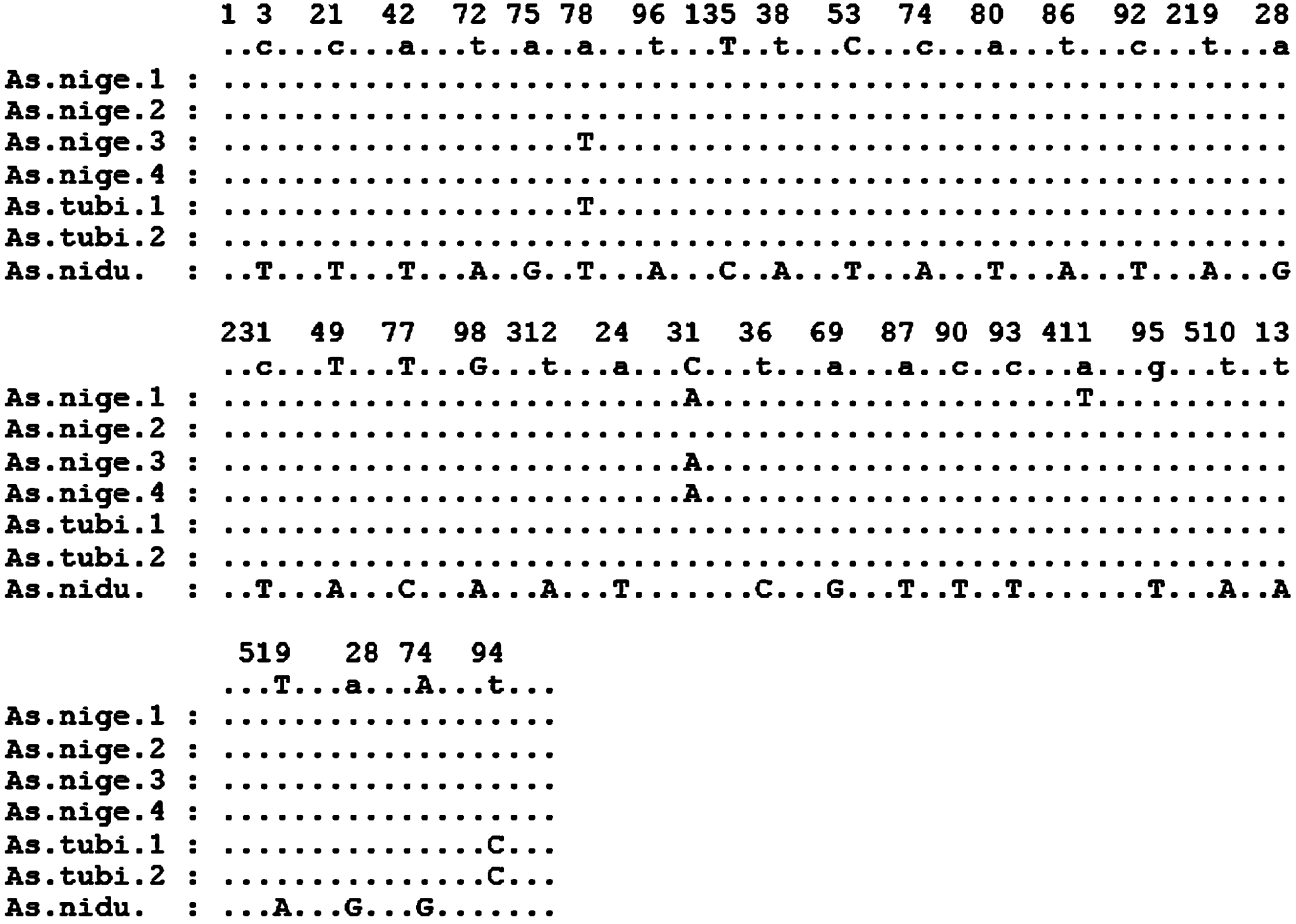
Nucleotide variation within the barcode region in seven strains *Aspergillus* from three closely related species. The GenBank Accession nos of the sequences used for generating the barcodes are AF178445 (As.nige.1), NC_007445 (As.nige.2), AF178444 (As.nige.3), AF178443 (As.nige.4), NC_007597 (As.tubi.1), AY802759 (As.tubi.2) and X00790(As.nidu). There are a total of 36 variant sites, consisting of 8 nonsynonymous sites (upper case in the consensus sequence) and 28 synonymous sites.

The foregoing analysis shows that standard DNA barcode sequences are sufficient to discriminate among a wide range of fungal species, although they do not contain enough sequence information to group these species into a reliable phylogeny. There is a second challenge for DNA barcoding studies, however, and that is the degree of sequence variability within a single species. We addressed this issue using the available data for multiple strains of the same fungal species. The database contains COI sequences from four strains of *Aspergillus niger* and from two strains of *Aspergillus tubingensis*. There are also sequences from three different strains of *C. neoformans*. The overall intraspecific distances are very small, both within and between the *Aspergillus* species. For example, in the barcode region, the predicted protein sequences are identical in the two strains of *A. tubingensis*, but there is one synonymous substitution at the DNA level (see Fig. 4). The average genetic distance among the four strains of *Aspergillus niger* was also very small (0.003) at both the protein and DNA level (see Table S1, Supplementary material), reflecting only three polymorphic sites in the barcode region resulting in one amino acid difference (see Fig. 4). Even though the interspecific distance between *A. niger* and *A. tubingensis* was also very small (0.004), strains of *A. niger* can still be distinguished from *A. tubingensis* at the DNA level using only the barcode region (see Fig. 4). For instance, at site 594, the four strains of *A. niger* contain a T nucleotide whereas the two *A. tubingensis* strains contain a C at this position (see Fig. 4). Thus, there would be no ambiguity in distinguishing these two species by using a character-based approach (DeSalle *et al*. 2005). DNA barcode sequences were also sufficient to group the three strains of *C. neoformans* together and to separate the two known varieties (Franzot *et al*. 1999); two of the strains of *C. neoformans* belong to one variety and the third strain to a different variety (Litter *et al*. 2005). Thus, based on the limited data available to date, DNA barcoding of fungi can both distinguish between different species and it can also group conspecific strains together.

**Fig. 5:**
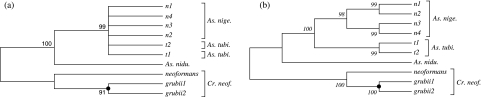
Extended barcode sequences have increased resolving power. Panel a shows an NJ tree constructed with the standard barcode sequence (∼600 bp). Panel b shows a tree based on an extended barcode sequence (∼1200 bp). • indicates the DNA sequences are identical in the two strains on that branch. Sequence accession nos are, AF178445 (n1), NC_007445 (n2), AF178444 (n3), AF178443 (n4), NC_007597 (t1), AY802759 (t2), X00790(As.nidu.), NC_004336 (grubii1), AY560510 (grubii2) and AY560610 (neoformans).

Although we can use diagnostic nucleotides at particular sites within the barcode sequence to distinguish between strains of *A. niger* and *A. tubingensis*, the low level of sequence divergence between the two species results in both being grouped together in the phylogenetic tree based on the standard barcode region (see Fig. 5a). This problem can be remedied, however, by extending the sequenced region from 600 to 1200 bp (see Fig. 5b). In other words, while sequences that are shorter than the standard barcode may suffice in many cases (Hajibabaei *et al*. 2006c), the converse is also true, that is in special cases of very low divergence between closely related species, an extended barcode sequence may be required.

## Discussion

Our results show that as the length of the molecular sequences is reduced, there is a concomitant loss of resolution at the internal nodes of the phylogenetic trees. This makes good intuitive sense. Despite this general loss of phylogenetic signal, however, the shorter sequences can still resolve the terminal nodes of the tree quite efficiently. This explains why DNA barcoding based on very short sequences has proved so successful for species identification (Hebert *et al.* 2004a, b; Hajibabaei *et al*. 2006a). By comparing the results presented in Figs 1–3, it is clear that there is, on average, a greater loss of signal at the deeper nodes in the tree, but this is not a universal rule; statistical support is retained for some of the deeper nodes even when using the short sequences. The key factor appears to be the relationship between the magnitude of the internodal distances and the age of the divergence. Deeper nodes can be resolved only when there is a large separation between the nodes but, on average, deeper nodes are less well resolved. At the other end of the timescale, DNA barcode sequences are also unsuitable for distinguishing between individuals within a population or between populations within a species. In this latter case, the divergence time is very recent, but the level of divergence between the short sequences is close to zero. Thus, at both ends of the timescale much longer sequences are required. In the case of species-level discrimination, however, the short sequences are sufficient. This is because the level of interspecies divergence tends to be large relative to the magnitude of the intraspecific variation (Hebert *et al*. 2003).

To address if other phylogenetic tree construction methods can be used to improve the loss in the bootstrap support in the barcode sequences, we compared trees constructed with different methods including different models in each method. When we used sequences from all five genes, we got results that are all very similar to what we found with the NJ K2P method. The results were also consistent with the recently published fungal phylogeny based on different molecular sequences (James *et al*. 2006). On the other hand, when we use the barcode sequences only, the statistical support for most of the internal nodes dropped sharply just as it did in the neighbour-joining trees (compare Figure S6 and Fig. 3a). This confirms that, although the method of analysis can have some effect on the trees obtained, the major effect is due to reduced sequence length in the barcode-based trees.

In addition to our general question about the minimum required sequence length for efficient species discrimination, we also wished to determine if the patterns of sequence variation in fungal mitochondria, and in the COI gene in particular, displayed similar patterns of variation to those observed in animals. This was by no means certain a priori since fungi have very different modes of reproduction from animals. Despite these differences between animals and fungi, the patterns of barcode variation are quite comparable between the two groups. Specifically, our results show that the phylogenetic trees constructed from longer mitochondrial sequences were consistent with accepted fungal phylogeny and with other trees constructed from molecular sequences (Bullerwell *et al.* 2003a, b; Kouvelis *et al*. 2004) and, in particular, the 5′end of the COI gene provided excellent resolution at the level of individual fungal species.

A possible complication in the use of mitochondrial barcodes for fungal species identification is the frequent occurrence of introns in fungal mitochondrial genes. This complication can be avoided, however, by amplifying mRNA using RT–PCR. This has recently been achieved for a region of the cytochrome *b* gene in plant pathogenic Basidiomycetes (Grasso *et al*. 2006).

Although our results show that a 600 nt sequence from the COI gene provides a useful DNA barcode for fungi, there is no reason that the barcode has to be exactly this length. Longer sequences will always perform as well or better, and we have shown that sequences half this length can also discriminate between most of the species. However, longer sequences are impractical for use in high-throughput screening programs and shorter sequences suffer from reduced resolving power, even at the species level. Based on this trade-off between sequencing effort and species discrimination, DNA barcodes that are less than a kilobase long will provide optimum results for most applications. In some cases, much shorter sequences may be sufficient as has recently been demonstrated by Hajibabaei *et al*. (2006c), while in other cases (such as where there are closely related sibling species) longer sequences may be required. As more data are collected, we should be able to fine-tune the barcode lengths for particular taxa.

In conclusion, we believe that DNA barcoding has great potential for use in the identification of fungal species. The barcode data can also provide a modest contribution to our knowledge about fungal phylogeny, and they can be included in studies of phylogenetic species recognition (Taylor *et al*. 2000). These latter applications will require extensive amounts of additional sequence information, however, in addition to the short barcode sequences.

## Supplementary material

The following supplementary material is available for this article:

Figure S1 NJ trees constructed with mitochondrial cox2 (A, left) cDNA sequence and (B, right) protein sequences. A1, Ascomycota Saccharomyces; A2, Ascomycota Schizosaccharomyces; A3, Ascomycota Sordariomycetes; A4, Ascomycota Eurotiomycetes; B, Basidiomycota; C, Chytridiomycota. The highlighted branches and subtrees indicate where the two trees differ in topology; the highlighted species indicated species was clustered differently in two trees.

Figure S2 NJ trees constructed with mitochondrial cox3 (A, left) cDNA sequence and (B, right) protein sequences. A1, Ascomycota Saccharomyces; A2, Ascomycota Schizosaccharomyces; A3, Ascomycota Sordariomycetes; A4, Ascomycota Eurotiomycetes; B, Basidiomycota; C, Chytridiomycota. The highlighted branches, subtrees and taxon indicate where the two trees differ in topology; the highlighted species indicated species was clustered differently in two trees.

Figure S3 NJ trees constructed with mitochondrial cob (A, left) cDNA sequence and (B, right) protein sequences. A1, Ascomycota Saccharomyces; A2, Ascomycota Schizosaccharomyces; A3, Ascomycota Sordariomycetes; A4, Ascomycota Eurotiomycetes; B, Basidiomycota; C, Chytridiomycota. The highlighted branches, subtrees and taxon indicate where the two trees differ in topology; the highlighted species indicated species was clustered differently in two trees.

Figure S4 NJ trees constructed with mitochondrial ATP6 (A, left) cDNA sequence and (B, right) protein sequences. A1, Ascomycota Saccharomyces; A2, Ascomycota Schizosaccharomyces; A3, Ascomycota Sordariomycetes; A4, Ascomycota Eurotiomycetes; B, Basidiomycota; C, Chytridiomycota. The highlighted branches, subtrees and taxon indicate where the two trees differ in topology, the highlighted species indicated species was clustered differently in two trees.

Figure S5 NJ trees constructed with mitochondrial cox1 barcode (A, left) cDNA sequence (∼600 bp) and (B, right) amino acid sequences encoded by the barcode DNA sequences. A1, Ascomycota Saccharomyces; A2, Ascomycota Schizosaccharomyces; A3, Ascomycota Sordariomycetes; A4, Ascomycota Eurotiomycetes; B, Basidiomycota; C, Chytridiomycota.

Figure S6 Maximum parsimony (MP) trees constructed by using (A, left) Close-Neighbor-Interchange (CNI) search method and (B, right) min-mini heuristic search method. A1, Ascomycota Saccharomyces; A2, Ascomycota Schizosaccharomyces; A3, Ascomycota Sordariomycetes; A4, Ascomycota Eurotiomycetes; B, Basidiomycota; C, Chytridiomycota.

Figure S7 NJ trees constructed with mitochondrial cox1 halfbarcode (A, left) cDNA sequence (∼300 bp) and (B, right) amino acid sequences encoded by the half-barcode DNA sequences. A1, Ascomycota Saccharomyces; A2, Ascomycota Schizosaccharomyces; A3, Ascomycota Sordariomycetes; A4, Ascomycota Eurotiomycetes; B, Basidiomycota; C, Chytridiomycota. The highlighted branches and sub-trees indicate where the two trees differ in topology, the highlighted species/taxa indicated species was clustered differently in two trees. • in panel b indicates the amino acid sequences are identical in the species on that branch.

Table S1 Genetic divergence of cox1 genes in different strains within Aspergillus niger and Aspergillus tubingensis species and between these two species.

This material is available as part of the online article from: http://www.blackwell-synergy.com/doi/abs/

10.1111/j.1471-8286.2007.01698.x

(This link will take you to the article abstract).
