# Supplementary material for: Assessing the effect of varying sequence length on DNA barcoding of fungi
Source: Mol Ecol Notes. 2007 May 1;7(3):365–73. doi: 10.1111/j.1471-8286.2007.01698.x (PMC1890918; doi:10.1111/j.1471-8286.2007.01698.x)
Supplement: Figure S1 — NJ trees constructed with mitochondrial cox2 (A, left) cDNA sequence and (B, right) protein sequences. A1, Ascomycota Saccharomyces; A2, Ascomycota Schizosaccharomyces; A3, Ascomycota Sordariomycetes; A4, Ascomycota Eurotiomycetes; B, Basidiomycota; C, Chytridiomycota. The highlighted branches and subtrees indicate where the two trees differ in topology; the highlighted species indicated species was clustered differently in two trees. [file men0007-0365-fs1.doc]

**Supplementary materials**

A B

**Figure s1.** NJ-trees constructed with mitochondrial cox2 (A, left) cDNA sequence and (B, right) protein sequences. A1, Ascomycota Saccharomyces; A2, Ascomycota Schizosaccharomyces; A3, Ascomycota Sordariomycetes; A4, Ascomycota Eurotiomycetes; B, Basidiomycota; C, Chytridiomycota. The highlighted branches and subtrees indicate where the two trees differ in topology; the highlighted species indicated species was clustered differently in two trees.

A B

**Figure s2.** NJ-trees constructed with mitochondrial cox3 (A, left) cDNA sequence and (B, right) protein sequences. A1, Ascomycota Saccharomyces; A2, Ascomycota Schizosaccharomyces; A3, Ascomycota Sordariomycetes; A4, Ascomycota Eurotiomycetes; B, Basidiomycota; C, Chytridiomycota. The highlighted branches, subtrees and taxon indicate where the two trees differ in topology; the highlighted species indicated species was clustered differently in two trees.

A B

**Figure s3.** NJ-trees constructed with mitochondrial cob (A, left) cDNA sequence and (B, right) protein sequences. A1, Ascomycota Saccharomyces; A2, Ascomycota Schizosaccharomyces; A3, Ascomycota Sordariomycetes; A4, Ascomycota Eurotiomycetes; B, Basidiomycota; C, Chytridiomycota. The highlighted branches, subtrees and taxon indicate where the two trees differ in topology; the highlighted species indicated species was clustered differently in two trees.

A B

**Figure s4.** NJ-trees constructed with mitochondrial atp6 (A, left) cDNA sequence and (B, right) protein sequences. A1, Ascomycota Saccharomyces; A2, Ascomycota Schizosaccharomyces; A3, Ascomycota Sordariomycetes; A4, Ascomycota Eurotiomycetes; B, Basidiomycota; C, Chytridiomycota. The highlighted branches, subtrees and taxon indicate where the two trees differ in topology, the highlighted species indicated species was clustered differently in two trees.

A B

**Figure s5.** NJ-trees constructed with mitochondrial cox1 barcode (A, left) cDNA sequence (~600 bp) and (B, right) amino acid sequences encoded by the barcode DNA sequences. A1, Ascomycota Saccharomyces; A2, Ascomycota Schizosaccharomyces; A3, Ascomycota Sordariomycetes; A4, Ascomycota Eurotiomycetes; B, Basidiomycota; C, Chytridiomycota.

A B

**Figure s6.** Maximum Pasimony (MP) trees constructed by using (A, left) Close-Neighbor-Interchange (CNI) search method and (B, right) Min-Mini Heuristic search method. A1, Ascomycota Saccharomyces; A2, Ascomycota Schizosaccharomyces; A3, Ascomycota Sordariomycetes; A4, Ascomycota Eurotiomycetes; B, Basidiomycota; C, Chytridiomycota.

A B

**Figure s7.** NJ-trees constructed with mitochondrial cox1 half-barcode (A, left) cDNA sequence (~300 bp) and (B, right) amino acid sequences encoded by the half-barcode DNA sequences. A1, Ascomycota Saccharomyces; A2, Ascomycota Schizosaccharomyces; A3, Ascomycota Sordariomycetes; A4, Ascomycota Eurotiomycetes; B, Basidiomycota; C, Chytridiomycota. The highlighted branches and sub-trees indicate where the two trees differ in topology, the highlighted species/taxa indicated species was clustered differently in two trees. ● in panel b indicates the amino acid sequences are identical in the species on that branch.

**Supplementary Table 1. Genetic divergence of cox1 genes in different strains within *Aspergillus niger* and *Aspergillus tubingensis* species and between these two species**

|  | Within *A. niger* (4 strains) | Within *A. tubingensis*  (2 strains) | Between *A. ngier* and *A. tubingensis* |
| --- | --- | --- | --- |
| cox1 protein | 0.006±0.002 | 0.000±0.000 | 0.010±0.004 |
| cox1 DNA | 0.006±0.002 | 0.001±0.001 | 0.018±0.003 |
| barcode protein | 0.003±0.002 | 0.000±0.000 | 0.004±0.004 |
| barcode DNA | 0.003±0.001 | 0.002±0.002 | 0.004±0.002 |
